# The Effects of Adequate Rest on Nurse Job Satisfaction, Burnout Prevention, and Physical Health in Medical and Emergency Units at a Hospital in Western Jamaica: Qualitative Study

**DOI:** 10.2196/84106

**Published:** 2026-01-23

**Authors:** Channon Smith, Keitumetse-Kabelo Murray, Natasha Croome

**Affiliations:** 1 Health Services Department, The Heart Foundation of Jamaica, 28 Beechwood Ave, Kingston 5, Kingston, Jamaica, +1876-581-3576; 2 School of Public Health, Faculty of Medicine, Imperial College London, London, United Kingdom

**Keywords:** emergency nursing, Jamaica, job satisfaction, nurse burnout, nurse-patient ratio, nurse well-being, qualitative research, rest, staffing shortages

## Abstract

**Background:**

The demanding work environment of nurses in medical and emergency units often results in high stress, job dissatisfaction, and burnout. Adequate rest is crucial for maintaining nurses’ physical health, mental clarity, and emotional resilience, yet it is often overlooked in these high-pressure settings. This qualitative study explores the perceptions of nurses at a hospital in Western Jamaica regarding the quality and duration of rest they receive and its impact on their professional, mental, physical, and personal well-being. The hospital was selected due to the unique challenges health care workers face in Jamaica, including limited resources, high patient loads, and frequent staff shortages, which may exacerbate rest-related issues.

**Objective:**

This study aimed to explore the perceptions of registered nurses working in the emergency and medical units of the hospital in Western Jamaica regarding their rest experience and its implications for burnout, job satisfaction, and overall health.

**Methods:**

The study used a constructivist epistemological lens and used purposive sampling to select 12 registered nurses. The principal researcher conducted in-depth interviews with each participant via Zoom, using a semistructured guide. Interviews lasted 25 to 45 minutes, were audio-recorded, and attended only by participants and the researcher. Thematic analysis was used to transcribe, code, and analyze the data, culminating in the development of a thematic map of findings.

**Results:**

The findings indicated that nurses face significant challenges in obtaining adequate rest due to staff shortages, heavy workloads, irregular shifts, and limited management support. A total of three primary themes emerged: (1) noncompliance with rest policies, (2) resource limitations, and (3) management issues, each influencing job satisfaction, burnout, and overall health. Within noncompliance, nurses highlighted suboptimal nurse-to-patient ratios, absenteeism, and inadequate break time. For example, ratios as high as “30 to 2” or “60 to 3” were cited, affecting nurses’ ability to take breaks. Resource constraints included inadequate staffing, insufficient staff replacement, and the absence of suitable rest areas. Management concerns included weak policy enforcement, inadequate policy awareness, and limited support for rest breaks. These challenges collectively contributed to poor sleep quality, increased stress, and diminished job satisfaction.

**Conclusions:**

The study highlights the need for systemic improvements to address nurse rest and well-being, including increased staffing, structured policies on break enforcement, and enhanced management engagement. While the study is specific to the hospital in Western Jamaica, the findings may have broader implications for health care systems in similarly resource-constrained settings in the Caribbean and other low- and middle-income regions.

## Introduction

Registered nurses (RNs) are among the most vital resources globally, a reality that was illuminated during the COVID-19 pandemic [1]. The pandemic not only highlighted the essential role that nurses play in maintaining health care systems but also brought global attention to the immense pressures they face [1]. As reported by the British Broadcasting Corporation, nurses were at the frontline, often working extended hours under extreme conditions, which magnified the importance of addressing issues such as adequate rest, burnout, and job satisfaction [2,3]. This growing global realization of the system relevance of the nursing profession continues to shape discussions on health care reforms and support for the nursing workforce.

While nurses are vital in delivering health care and patient care, there has been less focus on their health promotion. The demanding nature of nursing in medical and emergency units puts professionals at high risk for stress and burnout [3,4]. The World Health Organization defines burnout as emotional exhaustion from chronic workplace stress, leading to fatigue and decreased performance [5,6]. Reports of burnout among RNs are prevalent, particularly in high-pressure environments like the US health care system [7]. However, there is a noticeable gap in the literature regarding the role of rest in preventing burnout among nurses, particularly in Caribbean or low-resource contexts. Most existing research focuses on high-income countries, leaving a lack of context-specific understanding in regions such as Jamaica.

RNs are expected to provide high-quality care while managing intense workloads and irregular hours, underscoring the need for effective strategies to enhance nurse well-being and job satisfaction [7]. Job satisfaction reflects a positive emotional response to one’s role and work environment [6,8,9]. Among these strategies, ensuring adequate rest is a crucial yet often overlooked component, essential for physical recovery and maintaining mental clarity and emotional resilience [10,11].

Despite being an upper-middle-income country, Jamaica faces a low nurse-to-patient ratio that negatively affects the rest quality and job satisfaction of RNs [12]. In contrast, socio-economically similar countries like Cuba and the Dominican Republic have much higher ratios [12,13]. Given the significant role of rest in job satisfaction, burnout prevention, and overall health, there is a lack of comprehensive research on its effects in Jamaican health care settings. This study aims to fill this gap by examining the perceptions of nurses at the hospital regarding the quality and duration of their rest and its impact on their well-being. This study focuses specifically on the medical and emergency units due to their particularly high levels of stress and burnout, as well as practical access considerations. These wards also represent high-acuity environments where the effects of inadequate rest are likely to be most pronounced. In fact, burnout rates on these wards are notably higher, ranging from 25% to 55% [14,15].

Through an in-depth analysis of how rest influences nurse satisfaction and burnout, this research seeks to generate evidence that can drive meaningful improvements in health care work environments. By focusing on the specific context of Western Jamaica, an underresearched region facing high patient loads and limited staffing, this study offers context-specific insights into the impact of rest on nurse well-being and performance. Its findings aim to support the development of evidence-based policies and institutional practices that prioritize staff recovery, reduce burnout, and ultimately improve patient outcomes. Beyond contributing to academic understanding, this research aspires to influence workforce planning and retention strategies, offering actionable recommendations to help build a more resilient, efficient, and sustainable health care system in Jamaica.

## Methods

### Setting

The hospital, established in 1964, is a key facility in western Jamaica. As a type B hospital, it provides 5 basic specialties: general surgery, internal medicine, obstetrics and gynecology, orthopedics, and pediatrics, ensuring comprehensive care for its diverse population. Although its capacity is 190 patients, it currently houses up to 300, leading to significant overcrowding and challenges for the health care system and medical staff [16].

This was a qualitative study that used purposive sampling to identify research participants, grounded in a constructivist epistemological approach. This perspective recognized the coconstruction of knowledge between the researcher and participants, aligning with the study’s aim to explore the subjective experiences of nurses [17].

The principal researcher acknowledged their potential influence on the research process, particularly given prior acquaintance with 2 participants. Efforts were made to remain self-aware and neutral during interviews and analysis, to minimize bias and enhance the credibility of the findings.

### Participant Recruitment

Permission was obtained from the Director of Nursing Services at the hospital on April 8, 2024, after discussing the research’s objectives, methodology, and potential impacts. A formal written request outlining the research aims, methodology, ethical considerations, and data management was submitted on April 11, 2024. Following approval, a signed permission letter was issued. The hospital administration assisted in ethically disseminating recruitment emails to 15 potential participants (see Table 1 for inclusion/exclusion criteria). While all 15 acknowledged receipt, only 12 participated: 1 did not sign the consent form, and 2 failed to respond after signing.

**Table 1.** Inclusion and exclusion criteria.

Criteria	Inclusion	Exclusion
Employment	Registered nurses currently employed in the medical or emergency units at the hospital.	Nurses who do not work in the medical or emergency units at the hospital.
Clinical experience	Minimum of 1 year of clinical experience in their respective units.	Nurses with <1 year of clinical experience in their respective units.
Educational qualification	Possession of a Bachelor of Science degree in Nursing or a higher-level nursing qualification.	Nurses who do not possess a Bachelor of Science degree in Nursing or a higher-level nursing qualification.
Willingness to participate	Willingness to participate in qualitative interviews discussing their experiences and perceptions related to rest, job satisfaction, burnout, and physical health.	Nurses who are unwilling to participate in qualitative interviews discussing their experiences and perceptions related to rest, job satisfaction, burnout, and physical health.
Availability	Availability to participate in a 45‐ to 60-minute interview session, either in person or virtually.	Nurses who are not available to participate in a 45‐ to 60-minute interview session, either in person or virtually.
Language proficiency	Ability to understand and communicate in English effectively, as the interviews will be conducted in English.	Nurses who are unable to understand or communicate effectively in English, as the interviews will be conducted in English.
Male nurses	Male nurses who meet the above criteria are included, ensuring a diverse representation within the study.	Male nurses who do not meet the above criteria are excluded to maintain consistency in the participant pool and ensure a focused analysis.

### Data Collection and Analysis

An interview guide was developed with open-ended questions exploring nurses’ practices in managing rest, perceptions of rest’s impact on satisfaction and burnout, and the role of hospital policies in promoting well-being. It also included recommendations for improving rest in the medical and emergency units at the hospital (see Multimedia Appendix 1).

Prior to the interview, participants were emailed the participant information sheet and subsequently the consent form and were asked to sign them via DocuSign. Interviews, averaging 45 minutes, were all conducted via Zoom using a semistructured guide with only the participants and the principal researcher present. Each session was audio recorded for accuracy, with no photos or videos taken. Data from interviews were transcribed, coded, and analyzed thematically following the 6-step framework of Braun and Clarke [18], which involved familiarization with the data, generating initial codes, searching for themes, reviewing themes, defining and naming themes, and writing up. An inductive approach was used, allowing themes to emerge from the data without being driven by preexisting theories or frameworks [18].

The trustworthiness of the data was reinforced through transparency in the research process and ongoing critical reflection. While one researcher led the initial coding, all authors reviewed the coding framework and contributed to the interpretation of findings. This collaborative process helped ensure consistency, reliability, and analytical depth, supported by clear documentation maintained throughout. Feedback from all authors further shaped the development of the analysis and structure of the paper, enhancing its overall rigor and quality.

### Ethical Considerations

The research was conducted in adherence to ethical guidelines outlined by the Declaration of Helsinki and followed institutional protocols to ensure quality, integrity, and ethical responsibility [19]. The methodology was rigorously designed to ensure reliability, validity, and participant protection, following best practices for qualitative research [20].

Ethical approval was granted by the Research Governance and Integrity Team at Imperial College London (ethics application ID 7069331).

Data management was robust, with clear documentation and secure storage. Paper forms were scanned into the primary author’s OneDrive space and then securely shredded. The data were securely stored. Any sensitive data archived were encrypted and access was restricted to authorized personnel only. Risks were managed by transparent communication, voluntary participation, and anonymization of data. To anonymize the data, personal identifiers such as names were removed and replaced with pseudonyms. Unique codes were assigned to each participant, and any identifying information was stored separately from the research data to maintain confidentiality.

All participants received a Participant Information Sheet prior to recruitment and provided written informed consent electronically before taking part in the study. Participation was voluntary, and participants were informed of their right to withdraw at any time without consequence. No financial or material compensation was provided for participation.

## Results

### Participant Characteristics

Twelve RNs participated in the study (see Table 2). The mean age of the participants was 28 (SD 2.2; range 25‐33) years. All nurses held a Bachelor of Science in Nursing degree, with 1 nurse also possessing a critical care certificate.

Participants had a mean of 3.6 (SD 1.8; range 1.2‐6) years of experience working at the hospital. The sample was evenly distributed across clinical units, with 50% (6/12) of participants assigned to the Accident and Emergency (A&E) unit and 50% (6/12) to the Medical unit.

**Table 2.** Demographic and professional characteristics of nurses at the hospital: female participant 1‐11, male participant 12; numbers indicate the chronological order of interviews.

Pseudonyms	Age (y)	Educational background	Experience at hospital (y)	Current unit	Sex
Female Participant 1	29	BSN^a^	5	A&E^b^	Female
Female Participant 2	28	BSN	4.5	Medical	Female
Female Participant 3	33	BSN	5.5	Medical	Female
Female Participant 4	28	BSN	5	Medical	Female
Female Participant 5	30	BSN	3	Medical	Female
Female Participant 6	29	BSN	5	A&E	Female
Female Participant 7	27	BSN	1.5	A&E	Female
Female Participant 8	25	BSN	1.2	Medical	Female
Female Participant 9	29	BSN+critical care certificate	6	A&E	Female
Female Participant 10	27	BSN	3	A&E	Female
Female Participant 11	28	BSN	1	Medical	Female
Male Participant 12	32	BSN	2	A&E	Male

a BSN: Bachelor of Science in Nursing.

b A&E: Accident and Emergency unit.

### Themes

#### Overview

Three key themes emerged from the interviews, on factors influencing rest quality and its impact on job satisfaction, burnout, and physical well-being. These themes were as follows: (1) noncompliance with rest policies, with subthemes of high nurse-patient ratios, high absenteeism, and rest duration; (2) resources, including limited human resources and the absence of rest facilities; and (3) management, focusing on policy improvement and implementation, as well as nonadherence to duties (see Multimedia Appendix 2 for coding table).

#### Noncompliance With Rest Policies

##### Overview

Noncompliance with rest policies among nurses in public hospitals is a persistent and multifaceted issue that has serious implications for staff well-being and patient care. Although formal guidelines are in place to ensure that nurses receive adequate breaks during their shifts, various systemic challenges make it difficult to adhere to these policies. Three critical subthemes emerged in relation to this problem: high nurse-patient ratios, high absenteeism, and inadequate rest duration.

##### High Nurse-Patient Ratios

The issue of noncompliance with rest policies among nurses is exacerbated by unsustainable nurse-patient ratios, making it nearly impossible for nurses to take their designated 1-hour breaks. Participants reported ratios as high as “sometimes 30 to 2, 35 to 2, 60 to 3, it varies” (Male Participant 12: A&E), highlighting the overwhelming workload they face. It is also common for a single RN to manage a unit with only an enrolled assistant nurse, meaning that while the RN receives assistance, they are still solely responsible for the entire unit, including supervising the enrolled assistant nurse. The consensus was clear; without addressing these staffing imbalances, compliance with rest policies will remain a significant challenge.

##### High Absenteeism

High absenteeism among nurses is a significant consequence of noncompliance with rest policies, as many nurses report having various medical illnesses, feeling overwhelmed, and burnt out, leading them to take frequent sick days. One participant noted, “the call-in rate is very high because when you realize that you are burnt out and tired you’ll find that persons are not coming in” (Female Participant 1: Medical). Another participant echoed this sentiment, stating, “I will wake up in the morning and say, OK, yes, I’m going to make it to work today… I just find that I am tired, not just physically tired, but emotionally tired” (Female Participant 2: Medical). This chronic fatigue often results in nurses prioritizing their health over work obligations. The pervasive culture of exhaustion and the lack of adequate rest contribute to a cycle of absenteeism that further strains the already limited nursing staff, ultimately impacting patient care and overall hospital operations.

##### Rest Duration

Rest duration remains a significant issue among nurses in public hospitals in Jamaica. Although the policies mandate a 1-hour break during 8-hour day shifts and 2 hours for night shifts, these rest periods are rarely observed in practice. One participant verbalized, “You’re supposed to get one hour in the day shift and two hours in the night, but we don’t get that” (Female Participant 6: A&E). Participants highlighted that despite these official guidelines, the reality of high patient complexity and understaffing often makes it impossible to take the full allotted break or even time to eat. Many nurses expressed frustration with the gap between policy and practice, noting that the workload and staffing shortages leave little time for adequate rest. This chronic lack of rest not only exacerbates fatigue, medical illnesses, and burnout but also negatively impacts patient care [21,22].

### Resources

#### Overview

The availability and quality of resources, particularly human resources and physical infrastructure, play a critical role in shaping nurses’ ability to rest during their shifts. Inadequate resources contribute significantly to poor rest quality, increased burnout, and decreased job satisfaction. Two key subthemes emerged under this category: limited human resources and the absence of adequate rest facilities.

#### Limited Resources (Including Human Resources)

The theme of resources, particularly limited human resources, emerged as one of the main factors influencing rest quality and, consequently, job satisfaction, burnout, and physical well-being among nurses. Participants consistently expressed concerns about inadequate staffing levels, which directly impact their ability to take necessary breaks. Among all, 1 nurse articulated the challenge succinctly when asked if management does not actively hire new staff, stating, “If you go there now and say, oh, we need staff, they’re going to say based on the quota that they have… but be reminded they have opened a lot of different areas and the population has expanded” (Female Participant 9: A&E). The overwhelming workloads resulting from these staffing shortages leave little room for rest. The lack of adequate resources not only hinders compliance with rest policies but also exacerbates feelings of burnout, as nurses struggle to manage their responsibilities without sufficient support.

#### Absence of Adequate Rest Facilities

The lack of adequate rest facilities at the hospital severely affects nurses’ ability to recuperate during shifts. All participants expressed dissatisfaction with the current designated rest areas, citing issues such as overcrowding, noise from nearby units, and the combination of a bed and lunch area with a microwave in the same space, posing safety and health risks. While some nurses resorted to resting in their cars, others had no choice but to endure the suboptimal conditions. This shows that without proper rest facilities, nurses struggle to fully recover during their shifts, which in turn affects their physical well-being and their ability to provide quality patient care.

### Management

#### Overview

The role of hospital management, particularly nursing leadership, is central to ensuring that rest policies are effectively implemented and that nurses are supported in their demanding roles. However, participants highlighted ongoing management-related challenges that undermine nurse well-being and disrupt the delivery of quality care. Two key subthemes emerged: the need for policy improvement and implementation, and nonadherence to managerial duties.

#### Policy Improvement and Implementation

Participants expressed a clear need for more effective policies that not only address staffing levels but also prioritize the well-being of nursing staff. Among all, 1 participant noted, “I think we need more policies to actually not just cater for the staffing of the hospital… but also to cater to the nurses” (Female Participant 1: Medical). Despite the existence of policies that outline break times, the implementation of these policies is often lacking. As one nurse stated, “the policy exists… however, there’s no implementation of the actual policy” (Female Participant 7: A&E). This sentiment was echoed by another participant who remarked, “I don’t think there is a collaborative effort among the hospital, administration, and nurses in promoting nurse well-being through proper rest practices” (Female Participant 3: Medical). The need for management to actively engage in policy enforcement and to create a supportive environment for nurses is paramount, as inadequate rest not only affects nurse satisfaction but also compromises patient care and safety.

#### Nonadherence to Duties

The issue of nursing managers not adhering to their duties at the hospital has been a significant concern among the interviewees. Participants expressed frustration over the lack of support from nursing managers, particularly during critical times when the unit is short-staffed. One participant noted, “the sisters are supposed to come there and assist and ensure that the unit is running to full capacity… but you find that when you fall into an emergency situation… they either tell you that they are short-staffed or they tell you did you call this ward for this” (Female Participant 4: Medical). This lack of responsiveness leaves nurses feeling overwhelmed and unsupported, as they are often left to manage high patient loads and intense emergency situations without adequate assistance. Another participant highlighted that “most of their tasks, they leave it for the nurses to do while they basically do nothing” (Female Participant 9: A&E), indicating a perceived neglect of managerial responsibilities on the units. The absence of proactive engagement from nursing managers not only exacerbates the challenges faced by nurses but also compromises patient care and safety, as the staff is unable to effectively manage their duties under such conditions.

### Differences Based on Gender, Unit Type, and Experience Level

To further contextualize these findings, differences based on gender, unit type, and experience level were also observed among participants, offering deeper insight into how individual and situational factors shape nurses’ experiences with rest policy compliance.

#### Gender Differences

The male nurse often highlighted issues related to workload and understaffing with a strong focus on managerial support and policy enforcement. For example, the male participant in the A&E unit emphasized frustration with the lack of implementation of break policies. Female nurses frequently discussed challenges around balancing work demands with personal responsibilities, such as family care, which impacted their ability to rest adequately during shifts. They also noted more about the emotional toll and burnout symptoms.

#### Unit Differences (A&E vs Medical)

Nurses working in A&E units reported higher stress levels due to patient complexity and unpredictability of cases. They described fewer opportunities for breaks and greater difficulty taking rest because they were often the only RN on the unit during shifts. Nurses in the Medical units acknowledged the challenges of patient care but reported slightly more opportunities for breaks compared to A&E. However, they also noted the workload increased significantly during night shifts.

#### Experience and Role

More experienced nurses, working 3 years or more, tended to express frustration with systemic issues such as staffing policies and managerial accountability. Less experienced nurses, working less than 3 years, were more likely to discuss immediate physical fatigue and emotional exhaustion, focusing on day-to-day survival rather than broader systemic changes.

## Discussion

### Summary of Major Findings

This study examined the role of adequate rest in nurse satisfaction, burnout prevention, and physical well-being in medical and emergency units. It uncovered systemic challenges that hinder nurses from obtaining sufficient rest. High patient-to-nurse ratios, staffing shortages, and lack of managerial support were identified as key contributors to fatigue and burnout. Nurses’ personal responsibilities, such as caregiving at home, also affected their ability to prioritize rest. These findings show how both institutional and personal factors compromise nurses’ well-being and, in turn, patient care.

### Interpretation of Demographic Differences

Demographic differences reflect social, cultural, and professional dynamics shaping nurses’ experiences. Female nurses often balance work and caregiving roles, heightening stress and burnout risk [23]. A&E nurses reported higher fatigue levels than those in Medical units, due to the unpredictable nature of emergency care. Experienced nurses highlighted systemic issues, while newer nurses focused on the immediate physical and emotional toll, reflecting their frontline pressures [24].

### Interpretation of Findings

The study reaffirms the link between rest and well-being. Nurses with more rest reported better physical and mental health, supporting global research on this topic [25]. Challenges at this hospital, such as high workloads and weak managerial support, mirror trends in other resource-limited settings [26]. For example, a Namibian study also found burnout tied to understaffing and lack of rest infrastructure [27]. Nurses at the hospital in Western Jamaica frequently reported physical exhaustion and long-term health concerns, echoing global findings on rest-related health risks, including musculoskeletal pain and emotional exhaustion, key components of burnout [7,25,28].

### Relation to Wider Context and Integration With Existing Literature

Jamaica faces a severe brain drain, with 80% of skilled workers emigrating, including health care professionals [29]. Ranking second globally on the brain drain index [30], Jamaica’s workforce shortages worsen burnout. Similar trends are noted in Guyana and Trinidad and Tobago [31]. Cultural and economic pressures often lead nurses to work extended hours without adequate compensation or rest. In contrast, high-income countries enforce stricter work hour regulations. At Spanish Town Hospital in Southeast-Central Jamaica, the nurse-patient ratio is 1:10, far above ratios in wealthier nations, contributing to burnout and absenteeism [32-34]. The Maslach Burnout Inventory indicated “very high” burnout among nurses, especially in emotional exhaustion [22,35]. The International Council of Nurses [36] highlights the negative outcomes that occur from high-income countries attempting to address their nursing shortages through “inequitable international recruitment.” Through recruiting via migration, it leaves nursing workforces in low- and middle-income countries without adequate care, masks the underlying issues leading to high turnover, and costs low- and middle-income countries lost training expenses after public investment in education.

Nurses struggle to maintain work-life balance in these settings. Overcrowding and understaffing lead to long hours and little time for self-care [37,22]. The State of the World’s Nursing Report 2025 [38] illustrated that only 55% of countries had regulations on working hours and conditions, whereas the remaining 45% had partial or no regulations. Care packages for mental well-being of nurses were implemented in 42% of countries, whereas 64% only implemented partial or no care packages. A study in Iceland showed higher satisfaction among nurses working standard hours versus those on overtime [39]. However, extended shifts remain necessary at this hospital in Western Jamaica, negatively affecting health and morale. These findings call for urgent action to support nurses and improve patient care through systemic reform. This is supported by the International Council of Nurses 2025 report [36], which states that solutions such as “ensuring adequate staffing and a balanced skill mix and workforce capacity aligned with patients demands” need to be implemented.

### Strengths and Limitations

This study’s strength lies in its qualitative design, which captured rich personal narratives often missed in quantitative research [17]. Familiarity between the researcher and some participants may have encouraged openness. However, it may also have introduced bias. The lead researcher’s nursing background may have shaped interpretations. Additionally, excluding non-English-speaking nurses, such as Cuban staff [1], limited the diversity of views. With only 12 participants from 2 units, generalizability is limited. Self-reported data also carry risks of under- or over-reporting.

### Future Work

Future studies should explore strategies for staff recruitment and retention to reduce burnout. Research is needed on how managerial practices affect rest and well-being. Peer support systems and cultural change around rest and self-care should be evaluated. Post-COVID recovery efforts should prioritize mental health support and enforceable rest policies [40].

### Policy and Intervention Recommendations

The hospital should address high nurse-to-patient ratios by increasing staff, ensuring breaks without compromising care. California’s Nurse-to-Patient Ratios Law (1:2 in intensive care units; 1:4 in medical-surgical) reduced burnout and improved outcomes [4]. Structured break policies and cross-unit support systems are vital. UK hospitals implementing scheduled breaks reported reduced stress and higher job satisfaction [41]. Australia’s cross-unit model ensures continuity of care and relieves pressure during breaks [39,42,43].

Hospital leadership should prioritize nurse well-being. Programs in Australia train managers to promote self-care and regular breaks, leading to greater satisfaction and lower turnover [44]. At Cleveland Clinic in the United States, managerial training on rest and mental health improved morale and retention [45,46]. Wellness programs like Johns Hopkins’ Resilience in Stressful Events provide peer support and counseling, significantly reducing burnout and emotional exhaustion among nurses [47].

### Conclusion

This study underscores the role of adequate rest in preventing burnout and enhancing job satisfaction among nurses in medical and emergency units. The findings reveal that systemic barriers, such as high workloads, inadequate staffing, and chaotic work environments, significantly hinder nurses’ ability to achieve sufficient rest. Nurses in the emergency unit, in particular, face higher stress levels due to the demanding nature of their work, which worsens fatigue and burnout. The lack of localized studies focusing on the physical and mental well-being of Jamaican nurses, particularly in high-pressure emergency units, creates a significant academic gap.

Addressing these issues can enhance the global understanding of burnout in various contexts while providing region-specific strategies to improve nurse retention, job satisfaction, and overall health care quality in Jamaica. A multifaceted approach is required to tackle these challenges, incorporating policy changes, management training, and the creation of supportive work environments. By prioritizing the well-being of nurses through adequate rest, health care institutions can not only improve nurse satisfaction but also ensure better patient care outcomes.

## Supplementary material

10.2196/84106 Multimedia Appendix 1 Interview guide.

10.2196/84106 Multimedia Appendix 2 Coding table: from quotes to theme.

## Abbreviations

A&E: Accident and Emergency
RN: registered nurse
