# Supplementary material for: The Effects of Adequate Rest on Nurse Job Satisfaction, Burnout Prevention, and Physical Health in Medical and Emergency Units at a Hospital in Western Jamaica: Qualitative Study
Source: JMIR Nurs. 2026 Jan 23;9:e84106. doi: 10.2196/84106 (PMC12829585; doi:10.2196/84106)
Supplement: Multimedia Appendix 1 [file nursing-v9-e84106-s001.docx]

INTERVIEW GUIDE

Introduction:

Good day, I'm Channon Smith, a Public Health student at Imperial College London. My research focuses on the effects of adequate rest on nurse satisfaction, burnout prevention, and physical health within medical and emergency units at this hospital. Thank you for agreeing to participate in this interview. Before we begin, I want to ensure you're comfortable with recording this interview and using it for my research. You're free to stop the interview anytime, and you can also withdraw your data before it's fully transcribed and anonymised (which will happen within two weeks after our conversation). If you're okay with this, we can proceed with the interview.

Demographic and Professional Background:

Could you please share your age, educational background, and years of experience as a nurse at this hospital?

Current Practices in Rest Management:

How do you manage your rest time?

Perceptions of Rest and Well-being:

Can you share your understanding of how adequate rest impacts nurse satisfaction, prevents burnout, and influences physical health in medical and emergency units?

Do you believe there is a consensus among nurses regarding the importance of rest in relation to job satisfaction, burnout, and physical well-being?

a. If yes, what factors contribute to this consensus? If not, what variations in understanding do you observe?

Do you feel your views on the impact of rest align with those of your colleagues?

a. Please elaborate on your answer.

How do you perceive the implementation of rest management practices within medical and emergency units?

a. As a nurse, what steps do you take to promote adequate rest and well-being among your colleagues?

Roles in Promoting Nurse Well-being:

What role do hospital policies and administrative measures play in promoting and ensuring adequate rest for nurses in medical and emergency units?

How do nurses contribute to fostering rest and well-being among their peers within these units?

a. Can you identify factors that influence nurses' motivation to prioritise rest and well-being in their work environment?

Experiences and Effectiveness:

In your opinion, do the current methods and processes of promoting rest and well-being effectively address nurse satisfaction, burnout prevention, and physical health? Why or why not?

Do you believe there is a collaborative effort among the hospital administration, and nurses in promoting nurse well-being through proper rest practices? Please elaborate on your perspective.

Proposed Changes and Adaptations:

Do you think there should be adjustments to how rest and well-being are currently promoted within medical and emergency units?

a. If yes, what specific changes would you recommend? Please provide rationales for your proposed changes, including potential modifications to hospital policies, administrative roles, and nurses' practices related to rest and well-being.

Conclusion:

That concludes the interview questions. Is there anything else you'd like to discuss or share regarding this topic before we conclude the recording?
