# Supplementary material for: The Effects of Adequate Rest on Nurse Job Satisfaction, Burnout Prevention, and Physical Health in Medical and Emergency Units at a Hospital in Western Jamaica: Qualitative Study
Source: JMIR Nurs. 2026 Jan 23;9:e84106. doi: 10.2196/84106 (PMC12829585; doi:10.2196/84106)
Supplement: Multimedia Appendix 2 [file nursing-v9-e84106-s002.docx]

Coding Table: From Quotes to Theme

| Quotation | Initial Code | Subtheme | Theme |
| --- | --- | --- | --- |
| "Sometimes 30 to 2, 35 to 2, 60 to 3, it varies" (Male Participant 12: A&E) | Overwhelming patient load | High nurse-patient ratios | Non-compliance with rest policies |
| "The call-in rate is very high because when you realise that you are burnt out and tired..." (Female Participant 1: Medical) | Frequent absenteeism due to burnout | High absenteeism | Non-compliance with rest policies |
| "I will wake up in the morning and say... I am tired, not just physically tired, but emotionally tired" (Female Participant 2: Medical) | Emotional and physical exhaustion | High absenteeism | Non-compliance with rest policies |
| "You're supposed to get one hour in the day shift and two hours in the night, but we don’t get that" (Female Participant 6: A&E) | Break time not followed | Rest duration | Non-compliance with rest policies |
| "If you go there now and say, oh, we need staff... they have opened a lot of different areas and the population has expanded" (Female Participant 9: A&E) | Demand exceeds staffing levels | Limited human resources | Resources |
| "We don’t really have a proper resting place... just one room with a microwave, a bed and some chairs" (Female Participant 11: Medical) | Poor rest infrastructure | Absence of rest facilities | Resources |
| "The policy exists… however, there's no implementation of the actual policy" (Female Participant 7: A&E) | Policy not enforced | Policy improvement & implementation | Management |
| "We need more policies to actually not just cater for the staffing... but also to cater to the nurses" (Female Participant 1: Medical) | Need for nurse-centered policy | Policy improvement & implementation | Management |
| "The sisters are supposed to come... but you find that when you fall into an emergency... they either tell you that they are short-staffed or they tell you did you call this ward for this" (Female Participant 4: Medical) | Managerial absence in crisis | Non-adherence to duties | Management |
| "Most of their tasks, they leave it for the nurses to do while they basically do nothing" (Female Participant 9: A&E) | Perceived managerial neglect | Non-adherence to duties | Management |
